# Phenylpropanoid, Sapnol A, Lignan and Neolignan Sophorosides, Saposides A and B, Isolated from Canadian Sugar Maple Sap

**DOI:** 10.3390/molecules18089641

**Published:** 2013-08-12

**Authors:** Kazuko Yoshikawa, Sakiko Tani, Chihiro Baba, Toshihiro Hashimoto

**Affiliations:** Faculty of Pharmaceutical Sciences, Tokushima Bunri University, Yamashiro-cho, Tokushima 770-8514, Japan

**Keywords:** maple sap, *Acer saccharum*, sapnol, saposide, antioxidant activity, superoxide dismutase (SOD)

## Abstract

One new phenolic compound, sapnol A (**1**), and two new aromatic sophorosides, named saposides A (**2**) and B (**3**) were isolated from sugar maple sap. In addition, seven known phenolic compounds **4**–**10** were isolated. These structures were determined on the basis of NMR experiments as well as chemical evidence. Furthermore, all the isolated compounds **1**–**10** were tested for antioxidative activity by the superoxide dismutase (SOD)-like assay.

## 1. Introduction

**Figure 1:**
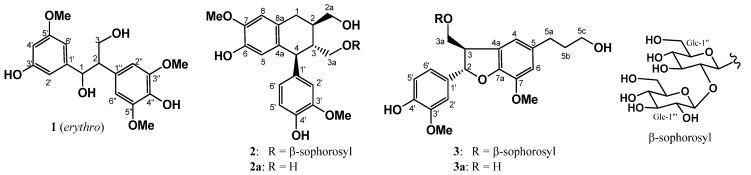
New compounds isolated in this work.

**Figure 2:**
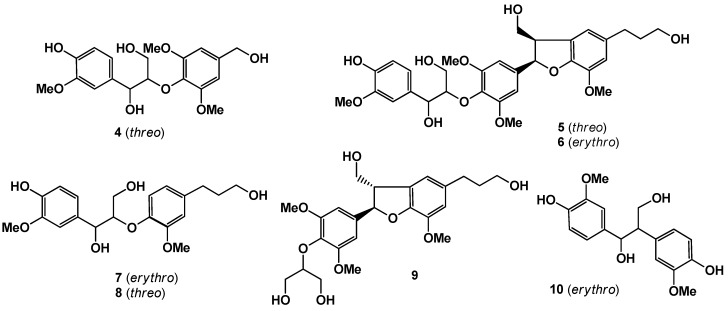
Known compounds isolated in this work.

Maple syrup is a premium natural sweetener obtained by concentrating maple sap collected from certain maple species, primarily *Acer saccharum* (Aceraceae), which is a tree native to North America [1]. Among the phytochemicals that have been previously reported from maple syrup, the phenolic components predominate [2,3,4]. The compounds present in maple syrup are possibly formed under the conditions of intensive heating involved in transforming sap to syrup. Thus, a comprehensive investigation of maple sap phenolics is necessary to evaluate the native components and biological properties of this natural sweetener. Previous phytochemical studies on maple sap were limited to the detection by gas chromatography of coumarin, vanillin, syringaldehyde, coniferyl aldehyde, and 2,6-dimethoxybenzoquinone [5]. Maple saps were passed through an Amberlite XAD, and then eluted subsequently at 20%, 50%, and 100% MeOH. The MeOH fraction that showed antioxidant activity at 15.5 μg/mL was subjected to bioassay-guided separation, fractionated by silica gel column chromatography, and further purified by reversed-phase HPLC to give three new compounds, designated as sapnol A (**1**), and saposides A (**2**) and B (**3**), along with seven known compounds, wilfordiol A (**4**) [6], *threo*-2-[4-[2,3-dihydro-3-(hydroxymethyl)-5-(3-hydroxy-propyl)-7-methoxy-2-benzofuranyl]-2,6-dimethoxyphenoxy]-1-(4-hydroxy-3-methoxyphenyl)-1,3-propanediol (**5**) [4], acernikol (**6**) [7], *erythro* -1-(4-hydroxy-3-methoxyphenyl)-2-[4-(3-hydroxy-propyl)-2-methoxy-phenoxyl)-1,3-propanediol (**7**), *threo* -1-(4-hydroxy-3-methoxyphenyl)-2-[4-(3-hydroxypropyl)-2-methoxyphenoxyl)-1,3-propanediol (**8**) [8], (+)-sakuraresinol (**9**) [9], and *erythro*-1,2-bis-(4-hydroxy-3-methoxyphenyl)-1,3-propanediol (**10**) [10] (Figure 1 and Figure 2). Here we describe the isolation, purification, and structural elucidation of **1**–**10** determined primarily by extensive NMR experiments, chemical degradation, and antioxidant activity by the superoxide dismutase (SOD)-like activity of all the isolated compounds.

## 2. Results and Discussion

**Figure 3:**
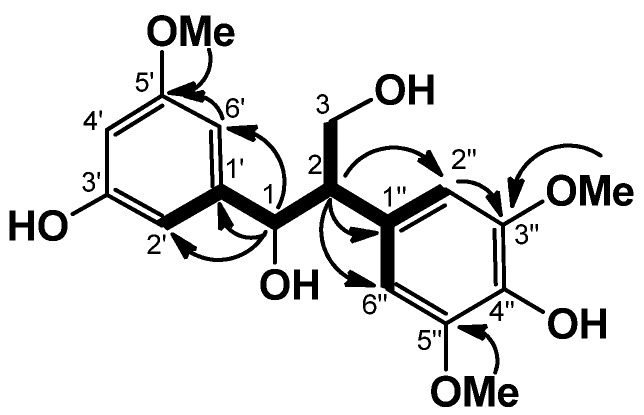
COSY (bold line) and selected HMBC (arrow line) of **1**.

Sapnol A (**1**) was obtained as an amorphous powder. HREIMS of **1** established the molecular formula C_18_H_22_O_7_, with an [M]^+^ peak at *m/z* 350.1349, indicating eight degrees of unsaturation. The UV spectrum of **1** showed absorption bands at 226, 280, and 307 nm, indicating the presence of a conjugated system. The IR spectrum of **1** suggested the presences of hydroxyl (3,395 cm^−1^) and aromatic ring groups (1,605, 1,520 cm^−1^). In the aromatic region, the ^1^H-NMR spectrum of **1** showed one set of ABC-type signals at δ 7.23 (1H, br. s), δ 7.24 (1H, br. s), and δ 7.31 (1H, br. s), an AA′-type signal at δ 7.03 (2H, br. s), and in the aliphatic region, three methoxy signals at δ 3.64 (3H, s) and δ 3.72 (6H, s), and one each of methine at δ 3.63 (1H ddd, *J* = 6.6, 6.3, 5.8 Hz), oxymethylene at δ 4.37 (1H dd, *J* = 10.6, 6.6 Hz), δ 4.58 (1H dd, *J* = 10.6, 5.3 Hz), and oxymethine at δ 5.73 (1H d, *J* = 5.8 Hz) (Table 1). The ^13^C-NMR spectrum revealed 18 signals. These were sorted, by HMQC data, into one methine carbon, three methoxy carbons, one oxymethylene carbon (δ 64.5), one oxymethine carbon (δ 74.8), and five tertiary and seven quaternary carbons due to two aromatic rings, and the presence of 1,3-propanediol could be revealed by the COSY correlations (Figure 3). The HMBC correlations from H-1 (δ 5.73)/C-1′, /C-2′, and /C-6′, from H-2 (δ 3.63)/C-1′′, /C-2′′, and /C-6′′, and NOEs between H-6′ (δ 7.23)/OMe (δ 3.64), and H-2′′ and H-6′′ (2H, δ 7.03)/OMe (6H, δ 3.72) could establish the substituted pattern of two aromatic rings which were connected by a 1,3-propanediol. The chemical shifts of C-1 (δ 74.8), C-2 (δ 57.4), and C-3 (δ 64.5) revealed that **1** is an *erythro* isomer [10]. From the above data, the structure of **1** was formulated as *erythro*-1-(3-hydroxy-5-methoxyphenyl)-2-(4-hydroxy-3,5-dimethoxyphenyl)-1,3-propanediol.

**Table 1:** NMR data for sapnol A (**1**) [600 MHz (^1^H) and 150 MHz (^13^C) in pyridine-*d*_5_].

Position	δ_C_	δ_H_ (mult, *J* in Hz)	Position	δ_C_	δ_H_ (mult, *J* in Hz)
1	74.8	5.73 (d, 5.8)	1′′	131.7	-
2	57.4	3.63 (ddd, 6.6, 6.3, 5.8)	2′′	108.6	7.03 (br. s)
3	64.5	4.37 (dd, 10.6, 6.6) 4.58 (dd, 10.6, 6.3)	3′′ 4′′	148.7 136.3	- -
1′	137.0	-	5′′	148.7	-
2′	111.7	7.31 (br. s)	6′′	108.6	7.03 (br. s)
3′	147.0	-	3′-OMe	55.8	3.64 (s)
4′	115.8	7.23 (br. s)	3′′-OMe	56.3	3.72 (s)
5′	148.3	-	5′′-OMe	56.3	3.72 (s)
6′	120.3	7.24 (br. s)			

**Figure 4:**
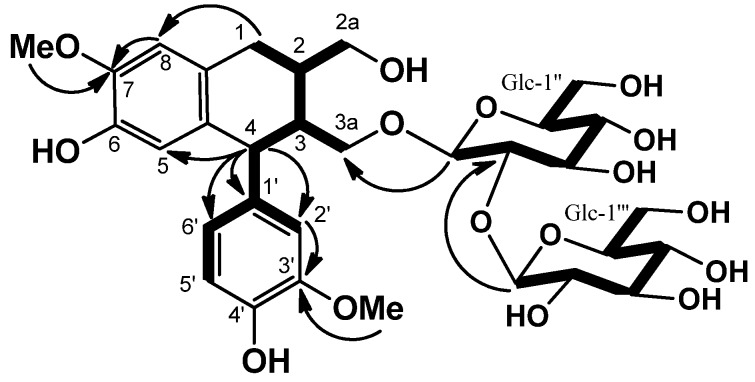
COSY (bold line) and selected HMBC (arrow line) of **2**.

Saposide A (**2**) was obtained as an amorphous powder, and showed an [M+Na]^+^ peak at *m/z* 707.2530 in HRFABMS, which corresponded to the molecular formula C_32_H_44_O_16_, requiring 11 degrees of unsaturation. The IR absorption bands at 3,530, 1,605 and 1,590 cm^−1^ were characteristic of hydroxyl and aromatic groups. The ^1^H-NMR and ^13^C-NMR spectra of **2** indicated the presence of two β-glucopyranosyl units [H-1′′: δ 4.79 (d, *J* = 7.8 Hz), C-1′′: δ 103.4, and H-1′′′: δ 5.38 (d, *J* = 7.7 Hz), C-1′′′: δ 106.4] [11]. On acid hydrolysis, **2** liberated D-glucose, identified by optical rotation using chiral detection in HPLC analysis [12]. The ^13^C-NMR spectrum revealed 20 signals due to the aglycone **2a**; these were sorted, by HMQC data, into one methylene carbon, three methine carbons, two methoxy carbons, two oxymethylene carbons (δ 63.4 and δ 67.9), and five tertiary and seven quaternary carbons due to two aromatic rings (Table 2). The planar structure of **2** was constructed using the 2D NMR spectra. Namely, analysis of the COSY spectrum led to three partial structures depicted by bold lines, which were connected on the basis of the long-range correlations observed in the HMBC spectrum (Figure 4). The HMBC correlations of H-1/C-8, of H-4/C-5,/C-1′,/C-2′, and /C-6′, of OMe (δ 3.73)/C-7, and of OMe (δ 3.70)/C-3′ revealed **2** to be an aryl-tetralin type lignan glucoside. The following NOEs between H-8/OMe (δ 3.73), and H-2′/OMe (δ 3.70) confirmed the substituent positions in two aromatic rings. The stereochemistry of **2** was also established by the NOEs between H-1β/H-3 and H-2/H-4, which were on the same face of H-2, H-4, and on the opposite face of H-3. The absolute configuration of the C-4 of **2** was established to be *S*, by a negative Cotton effect at 292 nm in the CD spectrum [13]. Thus, the aglycone **2a** could be identified with (+)-isolariciresinol. Further, the HMBC correlations of H-1′′ (δ 4.79)/C-3a (δ 67.9), and H-1′′′ (δ 5.38)/C-2′′ (δ 83.8) indicated that the β-sophorosyl moiety combined with C-3a position [11]. From the above findings, the structure of **2** was formulated as (+)-isolariciresinol-3a-*O*-β-sophoroside.

**Table 2:** NMR data for saposides A (**2**) and B (**3**) [600 MHz (^1^H) and 150 MHz (^13^C) in pyridine-*d*_5_].

Position	2		Position	3	
	δ_C_	δ_H_ (mult, *J* in Hz)		δ_C_	δ_H_ (mult, *J* in Hz)
1α 1β	32.4	3.19 (dd, 15.8, 4.7) 3.35 (dd, 15.8, 10.8)	2 3	88.6 52.1	6.09 (d,6.3) 4.12 (m)
2	38.2	2.71 (m)	3a	71.5	4.09 (m), 4.36 (m)
2a	63.4	4.20 (2H, m)	4a	129.2	-
3	44.1	2.39 (br t, 10.5)	4	117.7	6.92 (d, 1.2)
3a	67.9	3.64 (dd, 9.8, 3.4) 4.60 (dd, 9.8, 2.5)	5 5a	136.3 32.6	- 2.86 (2H, t, 6.4)
4	45.9	4.71 (d. 10.5)	5b	36.0	2.08 (2H, q, 6.4)
4a	133.3	-	5c	61.5	3.93 (2H, t, 6.4)
5	116.6	7.00 (s)	6	113.8	6.89 (d, 1.2)
6	144.9	-	7	144.6	-
7	145.7	-	7a	147.2	-
8	111.4	6.84 (s)	1′	133.6	-
8a	126.9	-	2′	111.1	7.37 (d, 1.8)
1′	136.6	-	3′	148.7	-
2′	113.4	7.40 (d, 1.8)	4′	148.1	-
3′	147.3	-	5′	116.5	7.19 (d, 8.1)
4′	145.1	-	6′	119.9	7.26 (dd, 8.1, 1.8)
5′	115.2	7.13 (d, 8.1)	7-OMe	56.3	3.83 (s)
6′	121.6	7.06 (d, 8.1, 1.8)	3′-OMe	55.9	3.67 (s)
7-OMe	54.8	3.73(s)	Glc-1′′	103.0	4.99 (d, 7.8)
3′-OMe	54.8	3.70 (s)	2′′	84.2	4.19 (m)
Glc-1′′	103.4	4.79 (d, 7.8)	3′′	78.1	4.21 (m)
2′′	83.8	4.15 (m)	4′′	71.5	4.24 (m)
3′′	78.4	4.28 (m)	5′′	78.6	3.93 (m)
4′′	71.5	4.24 (m)	6′′	62.5	4.38 (m) 4.53 (dd, 11.5, 2.4)
5′′	78.4	3.87 (m)	Glc-1′′′	106.6	5.27 (d, 7.7)
6′′	62.6	4.28 (m) 4.37 (dd, 11.5, 2.4)	2′′′ 3′′′	76.9 78.0	4.15 (m) 4.36 (m)
Glc-1′′′	106.4	5.38 (d, 7.7)	4′′′	71.2	4.24 (m)
2′′′	76.8	4.10 (m)	5′′′	78.6	3.93 (m)
3′′′	78.1	4.18 (m)	6′′′	62.5	4.38 (m)
4′′′	71.2	4.15 (m)			4.53 (dd, 11.5, 2.4)
5′′′	78.2	3.78 (m)			
6′′′	62.5	4.30 (m) 4.44 (dd, 11.5, 2.2)			

**Figure 5:**
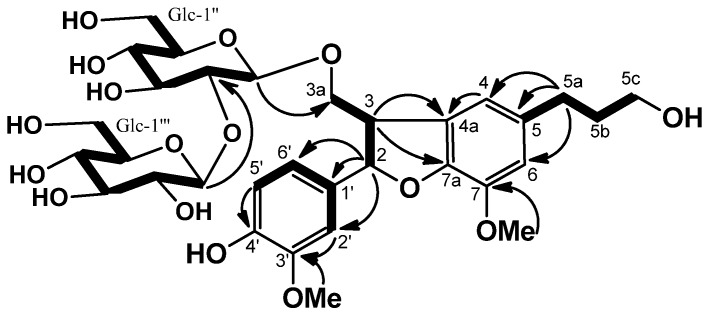
COSY (bold line) and selected HMBC (arrow line) of **3**.

Saposide B (**3**), obtained as amorphous powder, and was assigned the molecular formula C_32_H_44_O_16_, as exhibited by its HRFABMS (*m/z* 707.2522). The ^13^C-NMR spectrum of **3** showed the presence of one β-sophorosyl group [11]. On acid hydrolysis, **3** liberated D-glucose, identified by optical rotation using chiral detection in HPLC analysis. The ^13^C-NMR and HMQC spectra of **3** showed the signals of two methylene carbons, one methine carbon, two methoxy carbons, two oxymethylene carbons (δ 61.5 and δ 71.5), one oxymethine carbon (δ 88.6), and five tertiary and seven quaternary carbons due to the aglycone (**3a**), suggesting that **3a** is a dihydrobenzofuran-type lignan. The gross structure of **3** was determined by the same strategy as **2**. The COSY, HMBC correlations (Figure 5), and NOEs of OMe (δ 3.83)/H-6, and of OMe (δ 3.67)/H-2′ revealed **3a** to be 2,3-dihydro-2-(4-hydroxy-3-methoxy-phenyl)-3-(hydroxylmethyl)-7-methoxy-5-benzofuranpropanol. The NOEs between H-2 and H_2_-3a indicated the *trans* 2/3 configuration for **3**. The absolute configuration of C-2 was established to be *S*, since a negative Cotton effect at 226 nm was observed in the CD spectrum [14,15]. The location of the β-sophorosyl moiety at C-3a position was confirmed by the HMBC correlations of H-1′′ (δ 4.99)/C-3a (δ 71.5), and H-1′′′ (δ 5.27)/C-2′′ (δ 84.2). Consequently, the structure of **3** was concluded to be (2*S*,3*R*)-dihydrodehydroconiferyl alcohol-3a-*O*-β-sophoroside.

To the best of our knowledge, this is the first report of the isolation of known compounds **4**–**10** from maple sap. In this study, antioxidant activity of **1**–**10** was studied with an SOD assay kit. Vitamin C was used as a positive control. All isolated compounds showed moderate activities, with IC_50_ from 40 to 80 μM, and compound **1** exhibited marked SOD-like activity at IC_50_ 11.2 μM (Table 3).

**Table 3:** Antioxidant activity of **1**–**10**.

Compound	% Inhibition of at 100 μg/mL	IC_50_^b,^^d^
20% MeOH ^a^	38.2	918.9 ^c^^,^^d^
50% MeOH ^a^	66.2	17.5 ^c^^,^^d^
100% MeOH ^a^	67.3	15.5 ^c^^,^^d^
**1**	86.4	11.2
**2**	61.8	62.7
**3**	47.7	260.0
**4**	64.3	38.9
**5**	61.1	64.7
**6**	63.8	53.9
**7**	67.4	55.6
**8**	65.8	61.4
**9**	66.1	53.4
**10**	72.5	44.2
v. C ^e^	100	76.4

^a^ eluted fraction of maple sap; ^b^ IC_50_ values, in μM; ^c^ IC_50_ values, in μg/mL; ^d^ IC_50_ based on triplicate five-point titration; ^e^ v. C: ascorbic acid.

## 3. Experimental

### 3.1. General

Specific rotations and CD spectra were measured on a JASCO DIP-1030 and a JASCO J-725 polarimeter; UV spectra on a Shimadzu UV-1650PC; IR spectra, on a JASCO FT/IR-5300. NMR spectra were measured on a Varian Unity 600 spectrometer in C_5_D_5_N using TMS as an internal standard. FABMS was measured on a JEOL JMS-700 MStation. Column chromatography was carried out on silica gel (230–400 mesh; Merck, Darmstadt, Germany). Analytical TLC was performed on precoated Merck F_254_ silica gel plates and visualized by spraying with 30% H_2_SO_4_. HPLC was carried out on a JASCO PU-1580 pump equipped with a JASCO UV-970 detector and a COSMOSIL Pha column (5 μm, 20 mm i.d. × 250 mm; Nacalai Tesque, Kyoto, Japan).

### 3.2. Materials

Maple sap from *Acer saccharum* was collected at Quebec, Canada, in March 2011. A voucher specimen (TB 5429) is deposited in the Herbarium of Faculty of the Pharmaceutical Sciences, Tokushima Bunri University, Tokushima, Japan. The material was identified by one of the authors (K.Y.).

### 3.3. Extraction and Isolation

The maple sap (400 L) was passed through an Amberlite XAD-1180N column and eluted with 20% MeOH, 50% MeOH, and 100% MeOH. The MeOH fraction (19.5 g) was subjected to silica gel column chromatography with CHCl_3_-MeOH (9:1 → 0:10) to afford fractions 1–6. Fraction 2 (0.74 g) was purified by preparative HPLC (ODS, 55% MeOH) to afford wilfordiol A (**4**, 20.7 mg), and threo-2-[4-[2,3-dihydro-3-(hydroxymethyl)-5-(3-hydroxypropyl)-7-methoxy-2-benzofuranyl]-2,6-dimethoxy xyphenoxy]-1-(4-hydroxy-3-methoxyphenyl)-1,3-propanediol (**5**, 58.2 mg). Fraction 3 (2.0 g) was passed through silica gel with CHCl_3_-MeOH-H_2_O (25:8:0.1) and purified by preparative HPLC (ODS, 37% MeOH) to afford **1** (20.6 mg), acernikol (**6**, 33.1 mg), erythro-1-(4-hydroxy-3-methoxyphenyl)-2-[4-(3-hydroxypropyl)-2-methoxyphenoxy]-1,3-propanediol (**7**, 71.2 mg), threo-1-(4-hydroxy-3-methoxyphenyl)-2-[4-(3-hydroxypropyl)-2-methoxyphenoxy]-1,3-propanediol (**8**, 23.5 mg), and (+)-sakuraresinol (**9**, 15.5 mg). Fractions 4 (2.13 g), and 5 (1.7 g) were successively purified by preparative HPLC (ODS, 20%–50% MeOH) to afford erythro-1,2-bis-(4-hydroxy-3-methoxyphenyl)-1, 3-propanediol (**10**, 41.5 mg) from fraction 4, and **2** (11.5 mg), and **3** (56 mg) from fraction 5, respectively.

*Sapnol A* (**1**): An amorphous powder; [α]_D_^22^ −12.2° (*c* 1.6, MeOH); UV (MeOH) λ_max_ nm (log ε): 226 (4.01), 280 (3.61), 307 (3.43); FT-IR (dry film) ν_max_ cm^−1^: 3,395 (OH), 1,605, 1,520 (C=C), 1,070 (OH); ^1^H-NMR and ^13^C-NMR data (C_5_D_5_N) see Table 1; HREIMS *m/z* 350.1349 (Calcd. for C_18_H_22_O_7_, 350.1366).

*Saposide A* (**2**): An amorphous powder; [α]_D_^22^ +9.2° (*c* 0.6, MeOH); UV (MeOH) λ_max_ nm (log ε): 230 (4.06), 283 (3.76); CD (MeOH) nm (Δε): 292 (−6.30), 276 (+4.54), 239 (+4.55); FT-IR (dry film) cm^−1^: 3,530 (OH), 1,605, 1,590 (C=C); ^1^H-NMR and ^13^C-NMR data (C_5_D_5_N) see Table 2; HRFABMS *m/z* 707.2530 (Calcd. for C_32_H_44_O_16_Na, 707.2527).

*Saposide B* (**3**): An amorphous powder; [α]_D_^22^ −6.0° (*c* 0.8, MeOH); UV (MeOH) λ_max_ nm (log ε): 222 (4.09), 228 (4.10), 282 (3.75); CD (MeOH) nm (Δε): 294 (+0.67), 244 (+0.88), 226 (−1.67); FT-IR (dry film) λ_max_ cm^−1^: 3,380 (OH), 1,612, 1,520 (C=C), 1,035 (OH); ^1^H-NMR and ^13^C-NMR data (C_5_D_5_N) see Table 2; HRFABMS *m/z* 707.2522 (Calcd. for C_32_H_44_O_16_Na, 707.2529).

### 3.4. Acid Hydrolysis of ***2*** and ***3***

Each sample (1 mg) in 5% H_2_SO_4_-dioxane (1:1) was heated at 100 °C for 2 h. The reaction mixture was diluted with H_2_O, neutralized with Amberlite IRA-35 and evaporated *in vacuo* to dryness. The identification and the d or l configuration of glucose were determined using RI detection (Shimadzu RID-10A) and chiral detection (Shodex OR-1) by HPLC (Shodex RSpak NH_2_P-50 4D, CH_3_CN-H_2_O-H_3_PO_4_, 95:5:1, 1 mL/min, 47 °C), by comparison with an authentic sugar (10 mmol d-glc). The sugar portion gave the following peak of d-(+)-glucose at 20.6 min.

### 3.5. Superoxide Dismutase-Like (SOD) Activity Assay

SOD-like activity was determined according to the method of Ukeda [16] using a SOD Assay Kit-WST (Dojindo Lab., Kumamoto, Japan). A test sample was dissolved in DMSO to give a final DMSO concentration of 0.8% (v/v). 

## 4. Conclusions

The phenylpropanoid sapnol A (**1**), lignan sophoroside saposide A (**2**), and neolignan sophoroside saposide B (**3**) were isolated from sugar maple sap collected from *A. saccharum* (Aceraceae), together with seven known compounds, which included phenylpropanoids **4**, **10** and neolignans **5**–**9**. Additionally, all known compounds, **4**–**10** are reported for the first time from sugar maple sap. Some phenolics in maple syrup are possibly formed under the conditions of intensive heating involved in transforming sap to syrup. Here it should be noted that compounds **1**–**4**, **7** were not present in maple syrup. All isolated compounds showed moderate antioxidative activities (IC_50_; 40–80 μM). It was noted that compound **1** showed activity at IC_50_ 11.7 μM in this study. These results provide a potential explanation for the use of maple sap as a herbal medicine in the prevention of aging. Moreover, it should be noted that a number of compounds in maple sap remain unidentified.

## References

[1] Perkins T.D., van den Berg A.K. (2007). Maple syrup-pruduction, composition, chemistry, and sensory characteristics. Adv. Food Nutr. Res..

[2] Kermasha S., Goetghebeur M., Dumont J. (1995). Determination of phenolic compound profiles in maple products by high performance liquid chromatography. J. Agric. Food Chem..

[3] Li L., Seeram N.P. (2010). Maple syrup phytochemicals include lignans, coumarins, a stilbene and other previously unreported antioxidant phenolic compounds. J. Agric. Food Chem..

[4] Li L., Seeram N.P. (2011). Furher investigation into Maple syrup yields 3 new lignans, a new phenylpropanoid, and 26 ohter phytochemicals. J. Agric. Food Chem..

[5] Filipic V.J., Underwood J.C. (1964). Some aromatic compounds in sap composition of maple sap and syrup. J. Food Sci..

[6] Cao X., Li C.J., Yang J.Z., Wei B.X., Yuan S.P., Luo Y.M., Hou Q., Zhang D.M. (2012). Four new neolignans from the leaves of *Tripterygium wilfordii*. Fitoterapia.

[7] Morikawa T., Tao J., Ueda K., Matsuda H., Yoshikawa M. (2003). Medicinal foodstuffs. XXXI. Structures of new aromatic constituents and inhibitors of degranulation in RBL-2H3 cells from a Japanese folk medicine, the stem bak of *Acer nikoense*. Chem. Pharm. Bull..

[8] Miyase T., Ueno A., Takizawa N., Kobayashi H., Oguchi H. (1987). Studies on the glycosides of *Epimedium grandiflorum* MORR. var. *thunbergianum* (MIQ.) NAKAI. II.. Phytochemistry.

[9] Yoshinari K., Shimazaki N., Sashida Y., Mimaki Y. (1990). Flavanone xyloside and lignans from *Prunus Jamasakura* bark. Phytochemistry.

[10] Miki K., Takehara T., Sasaya T., Sakakibara A. (1980). Lignans of *Larix Leptolepis*. Phytochemistry.

[11] Yano I., Nishiizumi C., Yoshikawa K., Arihara S. (1993). Triterpenoid saponins from *Ilex integra*. Chem. Pharm. Bull..

[12] Yoshikawa K., Matsumoto K., Arihara S. (1999). New lanostanoid glycosides from the fruit body of *Laetiporus versisporus*. J. Nat. Prod..

[13] Otsuka H., Hirata E., Shinzato T., Takeda Y. (2000). Isolation of lignan glucosides and neolignan sulfate from the leaves of *Glochidion zeylanicum* (Gaertn) A. JUSS. Chem. Pharm. Bull..

[14] Matsuda N., Sato H., Yaoita Y., Kikuchi M. (1996). Isolation and absolute structures of the neolignan glycosides with the enantiometric aglycones from the leaves of *Viburnum awabuki* K. KOCH. Chem. Pharm. Bull..

[15] Machida K., Takano M., Kakuda R., Yaoita Y., Kikuchi M. (2002). A new lignin glycoside from the leaves of *Sambucus sieboldiana* (MIQ.) BLUME ex. GRAEBN. Chem. Pharm. Bull..

[16] Ukeda H., Kawana D., Maeda S., Sawamura M. (1999). Spectrophotometric assay for superoxide dismutase based on the reduction of highly water-soluble tetrazolium salts by xanthine-xanthine oxidase. Biosci. Biotechnol. Biochem..

